# Analysis of hospitalization expenses of 610 HIV/AIDS patients in Nantong, China

**DOI:** 10.1186/s12913-020-05687-4

**Published:** 2020-08-31

**Authors:** Xun Zhuang, Yujia Chen, Zunyou Wu, Sarah Robbins Scott, Renfei Lu, Zhengcheng Xu, Yuhui Yu, Wei Wang, Luyao Cao, Yuanyuan Liang, Gang Qin, Meiyin Zou

**Affiliations:** 1 Department of Epidemiology and Biostatistics, School of Public Health, Nantong University, Nantong, Jiangsu 226019 People’s Republic of China; 2 Nantong Center for Disease Control and Prevention, Nantong, Jiangsu 226007 People’s Republic of China; 3 National Center for AIDS/STD Control and Prevention, Chinese Center for Disease Control and Prevention, Beijing, 100872 People’s Republic of China; 4 Affiliated Infectious Disease Hospital of Nantong University, Nantong, Jiangsu 226000 People’s Republic of China

**Keywords:** AIDS, HIV, Hospitalization expense, Path analysis

## Abstract

**Background:**

The goal of this study was to describe the expenses related to human immunodeficiency virus (HIV) and acquired immune deficiency syndrome (AIDS) management and care in Nantong Infectious Disease Hospital from October 2013 through June 2017.

**Methods:**

The information of 610 HIV/AIDS inpatients were collected from the Electronic Medical Record System of the hospital. Univariate and path analysis were employed to evaluate the association between hospitalization expense and its related factors.

**Results:**

The average hospitalization expenses per person was 5454 RMB (Renminbi, the currency of China, about $808 USD) and 23,555 RMB (about $3489 USD), respectively for HIV/AIDS patients. The average length of hospital stay was 10.0 ± 5.5 days for HIV patients and 21.7 ± 12.4 days for AIDS patients. For HIV patients, laboratory test fees constituted 37.46% of total expenses; while drug fees accounted for the largest proportion for AIDS patients. Path analysis indicated that the length of hospital stay was the most important factor affecting total expenses (total path coefficient = 0.563 for HIV patients and 0.649 for AIDS patients). Total expenses for HIV-infected females was higher than that of males (total path coefficient = 0.217), and the more complications led to higher expenses for AIDS patients.

**Conclusions:**

Though antiretroviral therapy (ART) is provided for free in China, associated medical care, particularly hospitalizations and fees, continue to drive up the medical costs of patients living with HIV and AIDS. Understanding the factors influencing these costs are crucial for determining policies and strategies that can reduce the economic burden of HIV/AIDS patients in China.

## Background

Human immunodeficiency virus (HIV) and associated acquired immunodeficiency syndrome (AIDS) are global public health threats, due to their impact on the health and economic well-being of countries. Globally, more than 36 million people were living with HIV in 2017 and 1.8 million became newly infected with the disease in the same year [1]. In China, which is home to more than 1 billion people, the HIV epidemic continues to thrive. According to the National AIDS Epidemic Report of China, there were 758,610 HIV-infected cases in 2017 [2], more than double the number of cases (379,348) reported in 2010 [3]. More so, the disease was responsible for 26,787 deaths in the last year [2]. With the introduction of antiretroviral therapy (ART) however, treatment and management of HIV has drastically changed. In China, ART is provided for free to all diagnosed individuals, which can effectively inhibit virus replication and maintain a low level of viral load continuously, so as to prevent the spread of HIV and reduce the overall prevalence of HIV to less than 1% [3, 4]. The burden of disease however, or the death and loss of health due to HIV/AIDS or associated risk factors [5], continues to threaten the growth of China’s health and economic sectors. As patients live longer, the potential for other complications and risk factors arise, leading to costly medical care.

Financial access is a limiting factor for many people throughout China, especially for those living with a chronic illness such as HIV. Numerous studies have aimed to estimate the associated management costs for HIV-positive patients. In particular, studies have shown that complications of the disease can lead to impaired immune function, which in turn can increase hospitalization rates, hospital expenses, and patients’ financial burden [6, 7]. A recent report amongst HIV-positive patients in France published in 2018, showed that most patients presenting to the hospital with an opportunistic infection or at least one comorbidity had increased hospitalization costs [6]. Another study from South Africa showed that the average cost per HIV+ admission was $1783 United States Dollars (USD), with patients hospitalized, on average, for 9.3 days [7]. In China, the government implemented policies aimed at reducing the economic strain placed upon HIV-positive patients to manage their disease. The government implemented the ‘free antiretroviral treatment’ policy and the ‘appropriate reduction on expenditure of opportunistic infection treatment’ in 2004 [8], which reduced associated expenses for HIV/AIDS patients. Though the government provides free testing and ART to patients, the costs of other necessary procedures, such as diagnostic and laboratory tests and drugs for opportunistic infections, are not covered; thus the amount actually paid by the patient can still be quite high [9, 10].

Multiple studies in China have aimed to assess the associated medical costs of people living with HIV in high prevalence areas. One study amongst female AIDS patients estimated that associated hospitalization expenses reached 3419 RMB (Renminbi, the national currency of China, or about $515 USD), with drug costs to manage co-morbidities accounting for the largest proportion (48.54%) of expenses [11]. Another study in Yunnan Province in southern China found that the average cost of hospitalization for people living with HIV/AIDS was 2822 RMB (about $453 USD), of which the anti-toxicity reaction of highly active antiretroviral therapy (HAART) and anti-opportunistic infections were 882 RMB (about $141.6 USD) and 885 RMB (about $142.1 USD), respectively [10]. These studies however, have not accounted for the different costs of HIV-related care in high prevalence versus low prevalence areas. With limited budget to meet HIV service needs, questions around where best to allocate resources arise. Thus, as these previous studies assessed related cost of care in high prevalence areas, and the change of direct economic burden for AIDS was associated with the epidemic situation in the region [12], so we aimed to describe the costs of medical care and utilization of health services of HIV/AIDS patients residing in Nantong city in eastern China, a low-prevalence (0.42%) area with over 90% viral suppression. The results of this study can inform potential future financial planning of HIV/AIDS related policy in China.

## Methods

### Data collection

This was a retrospective analysis conducted in Nantong City from October 2013 to June 2017. All the laboratory-confirmed HIV or AIDS patients, meeting the definitive criteria for HIV/AIDS in accordance with the Diagnosis for HIV/AIDS of National Health Commission of the People’s Republic of China (2019) [13], who had been hospitalized at the Nantong Infectious Disease Hospital during the study period, were included. The Nantong Infectious Disease Hospital is the only hospital that treats HIV/AIDS patients in Nantong City. Medical records of the hospitalized patients were collected from the Electronic Medical Record System of the hospital. Members of the study team familiar with medical records and billing files used data collection forms to capture the following data: demographic characteristics, time of admission and discharge, disease diagnosis, hospitalization expenses, and clinical treatment records. Hospitalization expenses included the direct medical expenses of each patient, including costs of medication (medications for prevention and treatment of adverse events or opportunistic infections), laboratory tests, examinations, and medical service charges. Hospital expenditure data was collected from patient entry into the hospital until the end of their first hospital stay. For patients undergoing repeated hospitalizations, only data from their first admission was considered for this study. This study was approved by the Ethics Committee of Affiliated Infectious Disease Hospital of Nantong University. As this was a retrospective analysis of de-identified records data (i.e., no personally identifying information was collected), informed consent was not required.

### Calculation of expenses

The hospitalization expenses were extracted directly from the electronic billing system of the hospital. The patients’ hospital expenses during their first admission were divided into four categories: medications, laboratory tests, examinations, and medical service charges. Medications were those taken for the prevention and treatment of adverse events (ADEs) or opportunistic infections (OIs) only. We did not include the cost of antiretroviral therapy, as antiretroviral (ARV) drugs are provided for free in China. Laboratory tests were for diagnosing and monitoring patients’ condition and ARVs efficacy, such as HIV viral load tests, CD4/CD8 cell counts, hematologic parameters, liver and kidney function tests, urinalysis, C-reactive protein (CRP) tests, Epstein-Barr virus (EBV), and cytomegalovirus (CMV) tests. Examinations included radiologic examinations, such as chest X-rays, computed tomography (CT), electrocardiogram (ECG), magnetic resonance imaging (MRI), and abdominal ultrasound. Lastly, medical service charges included the costs associated with counseling, infusions, bed use, and medical supplies. Due to the long time span of this study, in order to make the costs comparable, we used the 2017 consumer price index (CPI) published by the National Bureau of Statistics as a benchmark to discount the costs over the years.

### Statistical analysis

Data were entered using Epi Data 3.1 and STATA version 14.0 software (STATA Corporation, College Station, Texas, USA) was used for all analyses. The *P*-value of less than 0.05 was considered as statistically significant. Patients were categorized into two groups: HIV patients (individuals who had not yet progressed to AIDS) and AIDS patients (patients who had progressed to AIDS) [13]. Standard descriptive statistics were used to describe the characteristics of HIV/AIDS patients. Normally distributed continuous data were expressed as means and standard deviations (SDs). The skewed hospitalization expenses data were expressed as median and inter-quartile range (IQR).

In uni-variate analysis, the factors influencing hospitalization expenses of HIV patients and AIDS patients were analyzed by Mann-Whitney U test (for 2 subgroups) and Kruskal-Wallis test (for multiple subgroups). Path analysis was used to describe the direct and indirect effects between factors and hospitalization expenses. The direct, indirect, and total path coefficients indicate the direct effect of one variable on the dependent variable, the indirect effect on the dependent variable through other variables, and the comprehensive effect. In the path analysis, the term “effect” refers to the statistical effect, not the causal effect. A direct effect was the factor that had an immediate impact on the outcome (hospitalization expenses), while an indirect effect was considered as any factor working through one or more intermediate variables, which impacted hospitalization expenses. The path analysis model was gradually fitted through multiple linear regression analysis. All study variables with *P* < 0.2 in the uni-variate analysis were included in the path analysis in order to investigate as many potential variables as possible. In our study, both the length of hospital stay and hospitalization expenses variables were highly skewed and regarded as endogenous variables, so they were converted into approximate normal distributions by natural logarithmic (ln) transformation (Table 1).

**Table 1:** Main factors and assignment table of hospitalization expenses

Variable code	Assignment	Value
Y_2_、Y_4_	ln (Hospitalization expense) (HIV), ln (Hospitalization expense) (AIDS)	Hospitalization expense
Y_1_、Y_3_	ln (Length of hospital stay) (HIV), ln (Length of hospital stay) (AIDS)	Length of hospital stay
X_1_	Actual age (years)	Age (years)
X_2–0_, X_2–1_	0:Male 1:Female	Gender
X_3–0_, X_3–1_, X_3–2_	0:MSM 1:Heterosexual 2:Other/unknown	Transmission route
X_4–0_, X_4–1_	0:200+ 1:<200	CD4 + T cell count at admission (cells/μl)
X_5–0_, X_5–1_, X_5–2_, X_5–3_	0:0 1:1 2:2 3: 3+	Number of complications

*HIV* human immunodeficiency virus, *AIDS* acquired immune deficiency syndrome, *MSM* men who have sex with men

## Results

### Demographic and clinical characteristics of 610 HIV/AIDS inpatients

A total of 642 cases were reviewed, of which 32 cases were excluded for missing baseline CD4 + T cell count data, leaving 610 cases included in the final analysis. Of the 610 patients, 142 were living with HIV and 468 had AIDS. Of those living with HIV, the male to female ratio was 2.9:1; the average age was 40.9 ± 13.2 years (range: 12–73 years), the median CD4+ T cell count at admission was 315.5 (IQR: 256.8–439.5) cells/μl; and the average length of hospital stay was 10.0 ± 5.5 days (range: 2–48 days) (Table 2).

**Table 2:** The demographic and clinical characteristics of 610 HIV/AIDS inpatients

Characteristics	HIV [n (%)] (*n* = 142)	AIDS [n (%)] (*n* = 468)
Gender		
Male	106 (74.6)	405 (86.5)
Female	36 (25.4)	63 (13.5)
Age groups (years)		
< 30	38 (26.8)	70 (15.0)
30–44	44 (31.0)	120 (25.6)
45–59	49 (34.5)	205 (43.8)
60–73	11 (7.7)	73 (15.6)
Transmission route		
MSM	27 (19.0)	46 (9.8)
Heterosexual	111 (78.2)	347 (74.2)
Other/unknown	4 (2.8)	75 (16.0)
Length of hospital stay (days)		
< 10	89 (62.7)	89 (19.0)
10–19	48 (33.8)	130 (27.8)
20+	5 (3.5)	249 (53.2)
CD4 + T cell count at admission (cells/μl)		
< 200	3 (2.1)	374 (79.9)
200+	139 (97.9)	94 (20.1)
Complications		
0	142 (100.0)	107 (22.9)
1	0 (0)	117 (25.0)
2	0 (0)	190 (40.6)
3+	0 (0)	54 (11.5)
ART		
Yes	22 (15.5)	135 (28.8)
No	120 (84.5)	333 (71.2)

*HIV* human immunodeficiency virus, *AIDS* acquired immune deficiency syndrome, *MSM* men who have sex with men, *ART* antiretroviral therapy

Of the AIDS patients, the male to female ratio was 6.4:1, the average age was 46.1 ± 13.0 years (range: 11–76 years), the median CD4+ T cell count at admission was 63.5 (IQR: 18.0–165.0) cells/μl; and the average length of hospital stay was 21.7 ± 12.4 days (range: 1–75 days) (Table 2). Generally, more AIDS patients were male, older, acquired their infection through heterosexual transmission, had longer hospital stays, and more complications than the enrolled HIV patients.

### Hospitalization expenses

From October 2013 to June 2017, people living with HIV in Nantong City spent an average of 5454 RMB (about $808 USD) for their related care, while AIDS patients had an average expense of 23,555 RMB (about $3489 USD), as seen in Tables 3 and 4. For HIV patients, the cost of laboratory tests accounted for the largest proportion of care, with approximately 2043 RMB (about $303 USD) in associated fees. Examination and medication expenses (1602 RMB and 1590 RMB, $237 USD and $235 USD, respectively) were the second and third most costly portions of care, while medical service charges accounted 219 RMB (approximately $32 USD) (Table 3).

**Table 3:** Hospitalization expenses for 142 HIV inpatients from 2013 to 2017

Year	2013		2014		2015		2016		2017		Total	
Hospitalization expenses	Median (CNY)	Mean (CNY)	Median (CNY)	Mean (CNY)	Median (CNY)	Mean (CNY)	Median (CNY)	Mean (CNY)	Median (CNY)	Mean (CNY)	Median (CNY)	Mean (CNY)
Medications	26,103	26,103	2438	6066	2671	6653	15	820	158	1256	121	1590
Laboratory tests	2834	2834	894	1546	1368	1799	2002	1980	2149	2196	2088	2043
Examinations	3221	3221	3706	2885	3402	3396	1111	1362	1025	1607	1115	1602
Medical service charges	804	804	260	654	535	476	46	147	46	232	46	219
Total	32,962	32,962	7407	11,151	8086	12,324	3325	4309	3560	5291	3489	5454

*HIV* human immunodeficiency virus, *CNY* Chinese Yuan

**Table 4:** Hospitalization expenses for 468 AIDS inpatients from 2013 to 2017

Year	2013		2014		2015		2016		2017		Total	
Hospitalization expenses	Median (CNY)	Mean (CNY)	Median (CNY)	Mean (CNY)	Median (CNY)	Mean (CNY)	Median (CNY)	Mean (CNY)	Median (CNY)	Mean (CNY)	Median (CNY)	Mean (CNY)
Medications	10,246	14,204	19,973	21,044	16,071	19,234	9740	12,762	3995	7265	12,688	15,391
Laboratory tests	2414	2768	2769	2872	2788	3020	2894	3180	2704	2844	2826	3015
Examinations	3589	3373	4340	4271	4293	4644	3917	4140	2932	3656	4041	4186
Medical service charges	398	789	618	844	700	967	804	1104	517	797	654	963
Total	17,465	21,134	27,289	29,031	24,573	27,865	17,315	21,186	9780	14,562	20,268	23,555

*AIDS* acquired immune deficiency syndrome, *CNY* Chinese Yuan

The cost of medications was the largest cost associated with care for AIDS patients. AIDS patients spent, on average, 15,391 RMB ($2280 USD) for medication related fees. Medical service charges was responsible for the smallest proportion of AIDS patients’ associated hospitalization costs, with approximately 963 RMB ($143 USD) (Table 4).

### Uni-variate analysis of hospitalization expenses

The uni-variate analysis showed that being a female and longer time spent in the hospital were factors related to higher hospitalization expenses (*P* < 0.05) among people living with HIV. For AIDS patients, the factors associated with higher hospitalization expenses were longer hospital stay, CD4+ T cell count < 200 cells/μl, and more complications (*P* < 0.05) (Table 5).

**Table 5:** Uni-variate analysis of hospitalization expenses for HIV and AIDS inpatients

Factors	HIV patients			AIDS patients		
	Median (IQR) (CNY)	H	*P-*Value	Median (IQR) (CNY)	H	*P-*Value
Gender		−2.288	0.022		1.251	0.211
Male	3372 (3025-4123)			20,914 (9155–33,332)		
Female	4133 (3238-8044)			17,616 (9837-27,135)		
Age groups (years)		5.683	0.128		0.607	0.895
< 30	3437 (3095-6365)			19,751 (8602-32,644)		
30–44	3328 (2942-4346)			21,426 (8903-35,004)		
45–59	3561 (3113-5060)			19,856 (10,366-32,935)		
60–73	5041 (3391-12,078)			19,269 (12,538-31,723)		
Transmission route		4.815	0.090		5.167	0.076
MSM	3272 (3113-3516)			14,409 (3569-31,872)		
Heterosexual	3561 (3025-6365)			20,329 (9708-33,558)		
Other/unknown	3406 (2690-5842)			21,477 (12,049-31,715)		
Length of hospital stay (days)		32.467	<0.001		253.559	<0.001
< 10	3265 (2916-3765)			5053 (3145-8875)		
10–19	3874 (3489-7203)			14,324(8791-18,890)		
20+	10,858 (10,446-26,053)			30,578(22,942-39,682)		
CD4 + T cell count at admission (cells/μl)		−0.929	0.353		−4.155	<0.001
< 200	3820 (3384-8052)			23,097 (9879-35,104)		
200+	3489 (3029-5060)			14,691 (8947-21,013)		
Complications		–	–		99.487	<0.001
0	3489 (3072-5060)			7668 (3944–16,911)		
1	–			17,982 (10,295–29,991)		
2	–			25,932 (17,265–36,988)		
3+	–			31,105 (20,336-37,533)		

*HIV* human immunodeficiency virus*, AIDS* acquired immune deficiency syndrome*, MSM* men who have sex with men, *IQR* interquartile range

### Path analysis of hospitalization expenses

**Fig. 1:**
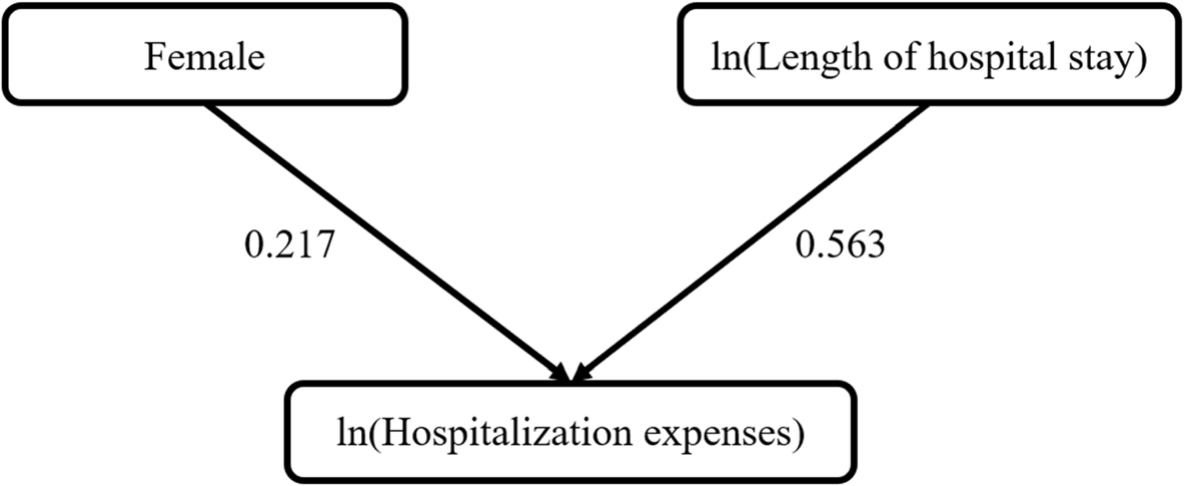
Path chart of hospitalization expenses for HIV patients

For HIV patients, the path mathematical model showed that the length of hospital stay had the strongest, direct, positive association with hospitalization expenses (total path coefficient = 0.563). Gender also had a direct, positive impact on hospitalization expense (total path coefficient = 0.217). Females’ hospitalization expenses was higher than that of males. However, there was no indirect effect on the hospitalization expenses (Table 6, Fig. 1). The coefficient of determination (R^2^) of the model was 0.569.

**Table 6:** Effect decomposition of factors on hospitalization expenses for HIV/AIDS inpatients

Factors	HIV patient					AIDS patient				
	Direct effect	Indirect effect	Total effect	Correlation coefficient with Y_1_	Correlation coefficient with Y_2_	Direct effect	Indirect effect	Total effect	Correlation coefficient with Y_3_	Correlation coefficient with Y_4_
ln (Length of hospital stay)	0.563	–	0.563	1.000	0.568^a^	0.649	–	0.649	1.000	0.727^a^
Female	0.217	–	0.217	0.020	0.228^a^	–	–	–	–	–
Complication = 1		–	–	–	–	0.241	−0.009	0.232	−0.076	−0.035
Complications = 2	–	–	–	–	–	0.359	0.062	0.421	0.195^a^	0.291^a^
Complications≥3	–	–	–	–	–	0.263	0.023	0.286	0.102^a^	0.172^a^

^a^ there is a correlation, the hypothesis test of the correlation coefficient (*P* < 0.05). *HIV* human immune deficiency virus, *AIDS* acquired immune deficiency syndrome

**Fig. 2:**
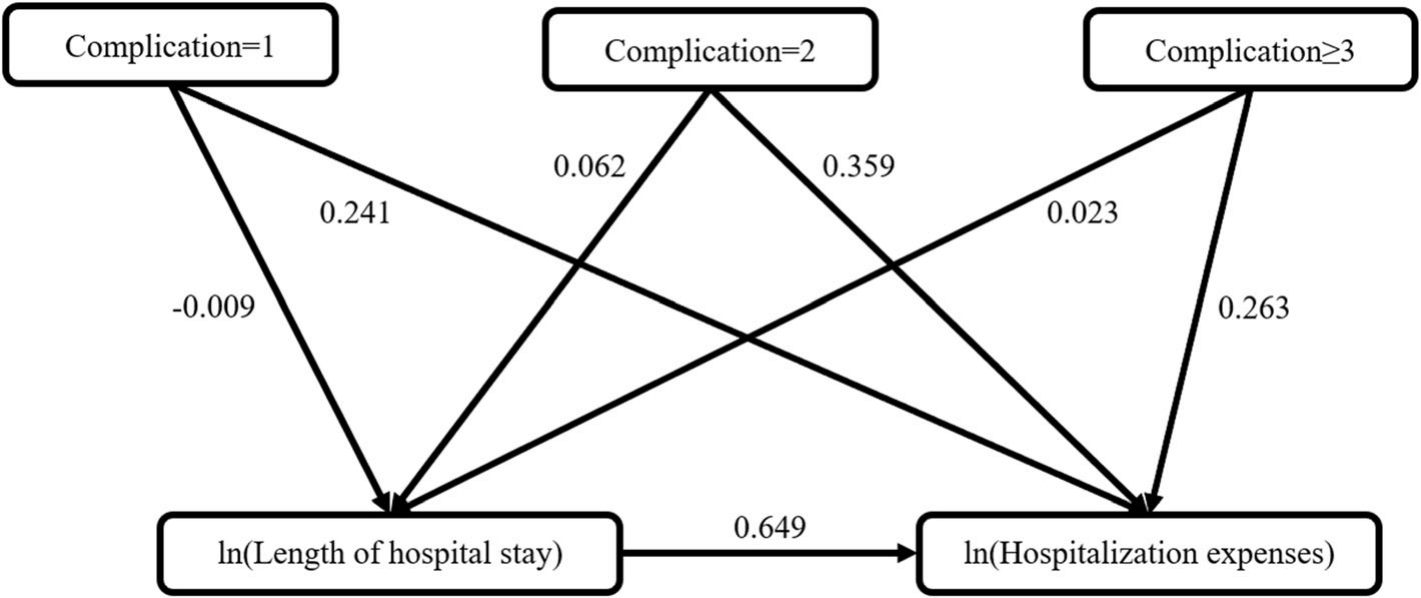
Path chart of hospitalization expenses for AIDS patients. The arrows represent the causal relationship, and the direction points to the result. The numbers are the path coefficient, which indicates the effect of the relevant variables on hospitalization expenses. Positive numbers indicate positive correlation and negative numbers indicate negative correlation

For AIDS patients, The path mathematical also found that the length of hospital stay had the strongest, direct, positive association with hospitalization expenses with a path coefficient of 0.649, while the number of complications had both direct and indirect impacts on hospitalization expenses (total path coefficient = 0.232, 0.421 and 0.286, respectively for the number of complication = 1, 2 and ≥ 3) (Table 6, Fig. 2). The coefficient of determination (R^2^) of the model was 0.613.

## Discussion

In our study, we described the HIV/AIDS-associated expenses of 610 inpatients in Nantong City. Though free antiretroviral therapy for HIV/AIDS patients was implemented in China at the end of 2004 [8], patients still accrued associated care costs. The average expenses per HIV patient was 5454 RMB (about $808 USD) for one hospital stay and 23,555 RMB (about $3489 USD) per AIDS patient for one hospital stay. Though Nantong is considered an economically developed area, HIV/AIDS can lead to detrimental impacts on resources, particularly if a positive person is unable to work due to his or her illness. Further research to estimate these indirect costs, as well as to understand how patients and their families are impacted by these healthcare costs, are needed.

A handful of factors were found to be associated with the increased hospitalizations costs amongst people living with HIV and AIDS in Nantong. For people living with HIV, gender directly affected hospitalization expenses. Females’ hospitalization expenses was 761 RMB (about $113 USD) more than that of males. The gender difference in hospitalization expenses may not be due to factors of gender itself, but perhaps to adherence to ART. Previous research has shown that females are more sensitive to symptoms, such as allergies or gastrointestinal reactions during ART, resulting in poorer adherence compared to males [14]. In turn, this can lead to more serious illness and higher hospitalization expenses for females. A follow-up assessment determining such factors associated with this higher cost could help inform gender-specific guidelines when assessing HIV-budgetary needs. This study also showed that for those living with HIV, the cost of laboratory tests accounted for the largest portion (37.46%) of associated in-patient costs, as those living with HIV may require semi-regular biochemical tests in order to track their disease progression while infected. Additional changes to current insurance policies, which promote reimbursement for laboratory tests could help lower patients’ financial burden and promote compliance [15].

In our study, the average hospitalization expenses of AIDS patients was 23,555 RMB (about $3489 USD), which is higher than a study in a hospital in Lanzhou, China reported of 15,714 RMB (about $2470 USD) [16]. The higher hospitalization expenses of AIDS in Nantong was related to the regional economic level and hospital grade [11, 12]. Nantong is located in an economically developed region in eastern China, which meant that local patients had greater health needs and stronger personal payment ability. Nantong Infectious Disease Hospital is a third-grade hospital, while the Infectious Hospital of Gansu Province, where the study in Lanzhou took place, is a second-grade hospital. Therefore, the higher grade the hospital, the larger expenditure patients will have to pay. Thus, not only should prevalence be considered when determining how best to allocate resources, but so should the economic viability of those infected. For AIDS patients, the cost of treatment for AIDS complications accrued a large economic burden [17], accounting for the largest portion of their expenses. This was consistent with other findings from Guangxi, China, which showed that the cost of medications constituted 65.33% of total expenses of AIDS infected patients [18]. Additionally, the number of complications not only directly affected hospitalization expenses, but also indirectly affected expenses through the length of hospital stay, indicating that the more complications or the more serious the condition an AIDS patient had, the more difficult to treat, leading to rising costs. In September 2015, the World Health Organization released updated treatment guidelines which recommended that ART be provided to all patients diagnosed with HIV, regardless of CD4+ T cell count [19]. Early detection and treatment to control viral load levels and improve CD4 + T cell counts are integral steps to reducing the risk of AIDS complications, and are thus, an ideal way to reduce the economic burden on patients and their families.

According to the findings from this study, the strongest predictor of higher hospital expenses was length of hospital stay (total path coefficient = 0.563 and 0.649, respectively for people living with HIV and AIDS patients). In our study the average length of hospital stay was 19.0 days, much higher than a study in South Africa, which reported an average length of stay of 9.3 days for HIV-positive admissions [7]. The average length of hospital stay is an important indicator, which can reflect the efficiency of hospitals in China. Without affecting the patient’s treatment, the hospital should take certain measures to reduce the length of hospital stay, which not only reduces the patient’s hospitalization expenses, but also improves the utilization rate of hospital medical resources. Expanded medical training of staff, as well as efforts to promote ART adherence may be preventive factors, which could help reduce medical expenditure in the long-term.

These findings should be considered in light of the study limitations. The cost in our study was lack of the fee of ART for its paid by the government. Participants in this study were limited to one infectious disease hospital in a low prevalence area in eastern China and as such, findings may not be generalizable to the other patients across the country. Additionally, in this study, we did not distinguish between the amounts paid by the patient and the amounts paid by their insurance companies. As such, further analysis addressing the true direct costs, supplemented with the potential impact on indirect costs, is needed. Further follow-up, which captures total hospitalizations costs of the patient, particularly out-of-pocket expenses and other associated costs of care not captured in this initial assessment, would help inform the true cost of HIV/AIDS care in Nantong City.

## Conclusions

Our study found that for HIV patients, laboratory fees was the largest contributor to expenses, while gender and length of hospital stay were the factors related to higher hospitalization expenses. For AIDS patients, associated drug fees was the most expensive part of care, and the number of complications and length of hospital stay had a significant impact on hospitalization expenses. Therefore, comprehensive measures such as early detection and treatment, promotion of ART adherence, efforts to shorten length of hospital stay, as well as reduce the associated laboratory and drug fees, are critical ways to reduce these costs and reduce the economic burden of the disease.

## Data Availability

The datasets used and/or analyzed during the current study are available from the corresponding author on reasonable request.

## Abbreviations

ADEs: Adverse events
AIDS: Acquired immunodeficiency syndrome
ART: Antiretroviral therapy
ARV: Antiretroviral
CPI: Consumer price index
CMV: Cytomegalovirus
CNY: Chinese Yuan
CRP: C-reactive protein
CT: Computed tomography
EBV: Epstein-Barr virus
ECG: Electrocardiogram
HAART: Highly active antiretroviral therapy
HIV: Human immunodeficiency virus
IQR: Inter-quartile range
MRI: Magnetic resonance imaging
MSM: Men who have sex with men
OIs: Opportunistic infections
RMB: Renminbi
SDs: Standard deviations
